# Boreal pollen contain ice-nucleating as well as ice-binding ‘antifreeze’ polysaccharides

**DOI:** 10.1038/srep41890

**Published:** 2017-02-03

**Authors:** Katharina Dreischmeier, Carsten Budke, Lars Wiehemeier, Tilman Kottke, Thomas Koop

**Affiliations:** 1 Bielefeld University, Faculty of Chemistry, Atmospheric and Physical Chemistry, D-33615 Bielefeld, Germany; 2 Bielefeld University, Faculty of Chemistry, Physical and Biophysical Chemistry, D-33615 Bielefeld, Germany; 3 Bielefeld University, Center for Molecular Materials, D-33615 Bielefeld, Germany

## Abstract

Ice nucleation and growth is an important and widespread environmental process. Accordingly, nature has developed means to either promote or inhibit ice crystal formation, for example ice-nucleating proteins in bacteria or ice-binding antifreeze proteins in polar fish. Recently, it was found that birch pollen release ice-nucleating macromolecules when suspended in water. Here we show that birch pollen washing water exhibits also ice-binding properties such as ice shaping and ice recrystallization inhibition, similar to antifreeze proteins. We present spectroscopic evidence that both the ice-nucleating as well as the ice-binding molecules are polysaccharides bearing carboxylate groups. The spectra suggest that both polysaccharides consist of very similar chemical moieties, but centrifugal filtration indicates differences in molecular size: ice nucleation occurs only in the supernatant of a 100 kDa filter, while ice shaping is strongly enhanced in the filtrate. This finding may suggest that the larger ice-nucleating polysaccharides consist of clusters of the smaller ice-binding polysaccharides, or that the latter are fragments of the ice-nucleating polysaccharides. Finally, similar polysaccharides released from pine and alder pollen also display both ice-nucleating as well as ice-binding ability, suggesting a common mechanism of interaction with ice among several boreal pollen with implications for atmospheric processes and antifreeze protection.

The formation of macroscopic ice crystals via nucleation and growth is of fundamental importance in various environmental systems such as in cloud, formation processes of Earth’s atmosphere^1^ and in cryobiological processes occurring in living organisms at subfreezing temperatures^2^,^3^,^4^,^5^. For example, intracellular ice crystal formation is usually considered lethal for the affected tissues. As a consequence, some insects, fish and plants produce antifreeze proteins which inhibit ice crystal growth in their body fluids through an adsorption-inhibition process. Hence, in the biosphere, crystal growth is often found to be the rate-limiting step for the formation of macroscopic ice crystals.

In the atmosphere, on the contrary, ice nucleation is normally rate-limiting for ice crystal formation. In deep-convective clouds and in cirrus clouds, ice nucleation does sometimes occur homogeneously, but often heterogeneous ice nucleation is the dominant pathway in mixed-phase clouds and in cirrus clouds^1^,^6^,^7^. Atmospheric heterogeneous ice nucleation is triggered by ice-nucleating particles, which traditionally were thought to consist predominantly of mineral dust particles^1^,^6^,^7^. However, recent field and laboratory measurements have stressed the importance of biological particles in the nanometre-to-micrometre size range for atmospheric ice formation^8^,^9^. Such biological particles include pollen, bacteria, microalgae, fungal spores and cellulose^10^,^11^,^12^,^13^,^14^,^15^. The concentration of biological and ice-nucleating particles were found to be enhanced during and after rainfall^14^, which may be due to ejection of soil surface matter after raindrop impaction^16^.

The physical basis for the distinction between high and low efficacy ice-nucleating particles is still a matter of intense research as are the properties and nature of so-called active sites, which are the locations on the particles where critical ice embryo formation occurs^17^,^18^,^19^. For some biological particles such as bacteria, the active site has been determined to be clusters of ice-nucleating proteins located in the cell membrane^2^. But also other types of ice-nucleating molecules such as (lipo)proteins and polysaccharides from bacteria, insects, lichen and plants have been known for several decades^2^,^20^,^21^,^22^,^23^,^24^,^25^. Such ice-nucleating molecules (INM) are not necessarily bound to the particles, but can also act as INM when separated from them, i.e., in the form of dissolved hydrated molecules or suspended molecular clusters. For example, Pummer *et al*.^26^ have shown that several types of pollen release INM when suspended in water: ice nucleation activity was maintained in the washing water filtrate devoid of original pollen particles.

Organisms have developed a large diversity of molecular compounds to sustain life at extreme conditions^27^. Interestingly, biological molecules such as proteins, glycoproteins and carbohydrates can interact with ice in sometimes counterintuitive ways. While some of these molecules act as ice nucleators as discussed above, others act by preventing ice crystal formation or by inhibiting the growth of existing ice crystals^2^,^3^,^4^,^5^,^25^,^28^,^29^, an ability that can be mimicked with some synthetic macromolecules^30^. The similarities and differences between ice-nucleating proteins and antifreeze proteins have been sought after for a long time^3^,^31^. While crystal structures of ice-nucleating proteins are not available, there is evidence that the central repeating units share similarities with those of some hyperactive antifreeze proteins, the principal difference being that ice-nucleating proteins are typically much larger than antifreeze proteins^3^,^32^,^33^,^34^,^35^. There is indeed very limited evidence that proteins or protein fragments show both functions. For example, an antifreeze glycoprotein from the rhizobacterium *Pseudomonas putida* was shown to also act as an ice-nucleating protein^36^. Interestingly, with a molecular weight of 164 kDa this glycoprotein is much larger than typical antifreeze (glyco)proteins with molecular weights between about 3 and 22 kDa. When 92 kDa of carbohydrate was removed from the antifreeze glycoprotein, its antifreeze activity was not affected but ice-nucleation activity was diminished. Thus it appears that the two opposing functions may be related to two different chemical moieties (carbohydrate and protein) within the antifreeze glycoprotein. Furthermore, it has been shown that a truncated 96-residue polypeptide section of an ice-nucleating protein from *Pseudomonas syringae* also exhibits ice-binding ‘antifreeze’ ability by showing both ice shaping and thermal hysteresis^37^. In light of this discussion, we wondered whether birch pollen and other types of pollen also contain molecules with ice-binding ‘antifreeze’ properties in addition to the recently observed presence of ice-nucleating molecules with a molecular weight of about 100–300 kDa^26^,^38^. Hence, we performed experiments specific to the analysis of typical antifreeze properties such as ice shaping and ice recrystallization inhibition and we also characterized the ice-nucleating properties in more detail.

## Results

### Ice nucleation experiments

**Figure 1:**
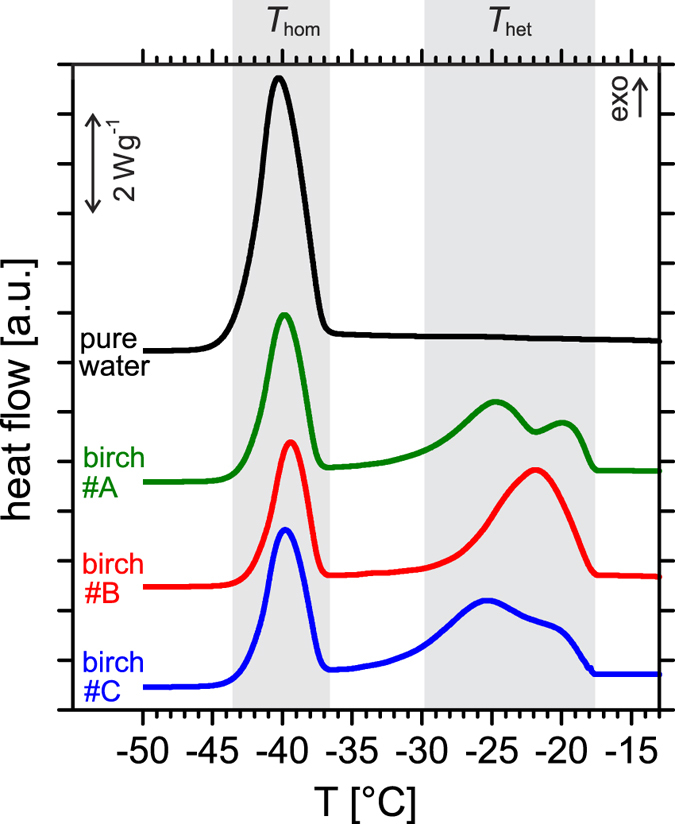
Heterogeneous ice nucleation triggered by pollen INM. DSC thermograms displaying homogeneous (*T*_hom_) and heterogeneous (*T*_het_) ice nucleation signals in emulsified droplets of pure water (black) and washing water of batches #A, #B, and #C of birch pollen (green, red, and blue, respectively). All experiments were performed at a cooling rate of −5 °C min^−1^.

In order to fully characterize the properties of birch pollen INM from *Betula pendula* we performed ice nucleation experiments with microliter-sized water droplets using an optical freezing array (BINARY) and with picoliter-sized droplets using differential scanning calorimetry (DSC), see Methods. The results of the microliter-sized droplet experiments agree closely with previous measurements^12^,^39^ suggesting that about 10^4^ INM are released per individual pollen particle, see Supplementary Fig. S1. For the DSC experiments, we prepared inverse (water-in-oil) emulsion samples, in which the picoliter-sized droplets contained either pure water or birch pollen washing water, see Fig. 1. In pure water droplets, only one exothermic peak representing homogeneous ice nucleation was observed at *T*_hom_ ≈ −38 °C (black line in Fig. 1), in agreement with experimental homogeneous ice nucleation rates^40^. When birch pollen washing water (WW) was investigated, either one or two additional peaks appeared at *T*_het1_ ≈ −18 °C and *T*_het2_ ≈ −22 °C (green, red, and blue lines in Fig. 1), indicating heterogeneous ice nucleation induced by two ice nucleators of different efficacy. These observations are consistent with previous measurements on heterogeneous ice nucleation of different types of birch pollen and birch pollen washing water^12^,^26^,^38^,^39^. Moreover, we studied the effect of additional solutes such as glucose or NaCl on the INM contained in birch pollen washing water (see Supplementary Fig. S2). The experimental results suggest that the ice-nucleating ‘active sites’ of birch pollen INM do not undergo significant solute-induced chemical or structural modification, in agreement with the behaviour of ice-nucleating proteins from *Pseudomonas syringae*^41^,^42^.

### Ice affinity purification

**Figure 2:**
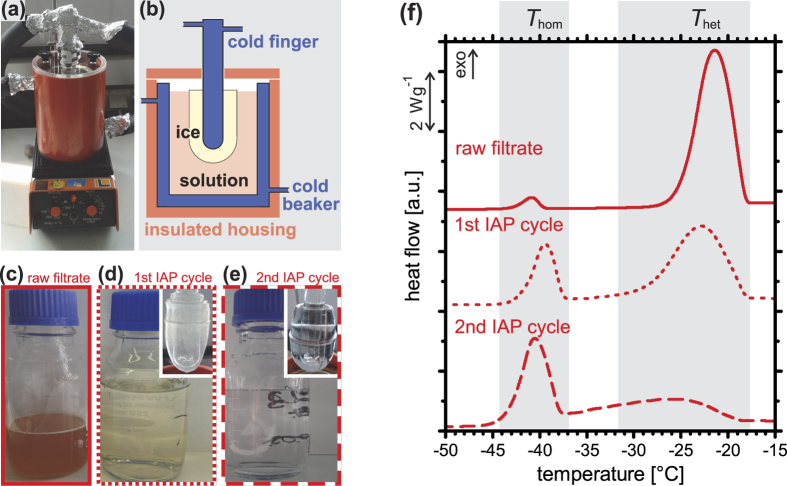
Ice affinity purification of birch pollen washing water. (**a**) Photograph and (**b**) schematic drawing of the experimental setup. (**c**) Raw birch pollen #B washing water filtrate. (**d**) Solution obtained from the first round of ice affinity purification (IAP) after melting the ice (inset) grown from raw filtrate. (**e**) Solution (and ice) after second IAP cycle. (**f**) DSC thermograms of emulsified samples of solutions shown in (**c**), (**d**) and (**e**), all displaying a homogeneous ice nucleation signal at about −38 °C (*T*_hom_) and a heterogeneous ice nucleation signal at about −18 °C (*T*_het_).

In order to investigate whether or not any ice-binding molecules are contained in birch pollen washing water, we removed other soluble components released from the pollen and, thus, contained in the washing water using ice affinity purification (IAP). This technique has been developed for purification of antifreeze proteins, i.e., molecules that bind to ice, from other non-binding proteins and solutes^43^. We followed the basic principle of the previously reported method^43^, see Fig. 2a,b and Methods. The raw filtrate solution (Fig. 2c) was placed into a double-walled glass beaker cooled to 0.0 ± 0.1 °C using a temperature-controlled circulating bath and an insulated housing. A glass cold finger was cooled to slightly below the ice melting temperature and immersed in pure 0 °C water containing some ice crystals for 5 min until a thin layer of ice had formed on the cold finger surface. Thereafter the cold finger was placed into the birch pollen raw filtrate solution and slowly cooled starting somewhat below the ice melting temperature (−0.7 ± 0.1 °C in the first and −0.3 ± 0.1 °C in the second cycle) at a rate of −0.1 °C and −0.05 °C per hour, respectively, until a large ice section had formed after one to two days (inset to Fig. 2d). This ice was removed and melted (Fig. 2d) into a new beaker and this solution IAP1 was used for a second run of ice affinity purification resulting in another solution sample IAP2 (Fig. 2e). Using this procedure, most solutes are expelled from growing ice crystals as indicated by the colourless solution after two IAP cycles (Fig. 2e) when compared to the brownish raw filtrate (Fig. 2c) and by an increase in ice melting temperature of ~0.5 °C. In contrast, molecules that bind to ice can be built into the slowly growing ice crystals^3^,^43^. A comparison of the heterogeneous ice nucleation peak in the DSC thermograms before and after ice affinity purification (see Fig. 2f) indicates that birch pollen INM are contained in the IAP solutions, albeit at reduced concentration. These experiments are an indication for the ability of birch pollen INM to bind to ice crystals in a manner similar to antifreeze proteins. Therefore, we performed additional experiments to investigate whether the ice affinity-purified sample does also show other hallmarks of antifreeze activity, such as ice shaping, ice recrystallization inhibition and thermal hysteresis, see below.

### Ice-shaping experiments

**Figure 3:**
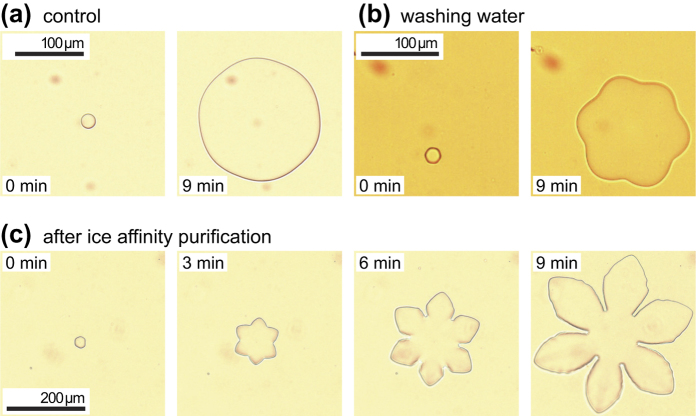
Microphotographs of ice crystals growing in birch pollen washing water. (**a**) Ice crystal growth in pure sucrose solution (control). (**b**) Ice crystal growth in raw filtrate of birch pollen #A washing water after heating to 90 °C for three hours to increase concentration. (**c**) Consecutive microphotographs of an ice crystal growing in the presence of birch pollen #B washing water after two ice affinity purification cycles; see also the entire sequence in Supplementary Video S1. In all experiments very small cooling rates of −0.01 °C min^−1^ were applied at temperatures just below the ice melting temperature to ensure that shaping originates from interaction with ice-binding molecules^4^,^44^. Note that while the absolute size and growth rate of the crystals in the three experiments are not directly comparable due to variable starting conditions (size, solute concentration, temperature etc.), the shape of each crystal is representative for each experiment.

Ice crystals growing slowly in aqueous solutions of non-ice-binding solutes (e.g., sucrose) just below the ice melting temperature show a round shape as this reduces the crystal’s total surface area and thus its overall interface energy (Fig. 3a)^4^,^44^. Ice crystals growing in birch pollen washing water, however, resulted in hexagonal ice crystals that grew into a ‘flower-shaped’ morphology (Fig. 3b), which is a strong indicator for the presence of ice-binding molecules. The hexagonal shape was observed even after heating the washing water for 3 hours at 90 °C, suggesting that these ice-binding molecules are non-proteinaceous. Infrared spectra, see below, suggest that they are polysaccharides. After two rounds of ice affinity purification and evaporating some of the water at reduced pressure at about 20–30 °C to increase the concentration, flower-shaped ice crystals were obtained (Fig. 3c). These grew very slowly over the course of several minutes when cooled at a rate of −0.01 °C min^−1^, see also the corresponding Supplementary Video S1. Together, these observations clearly indicate that the investigated ice-affinity purified molecules interact and bind to ice in a way identical to antifreeze proteins. We note that similar ‘flower-like’ ice crystal shapes were observed previously for antifreeze proteins derived from plants, e.g., winter rye glucanases and *Hippophae rhamnoides* chitinases^45^,^46^.

### Ice recrystallization inhibition

**Figure 4:**
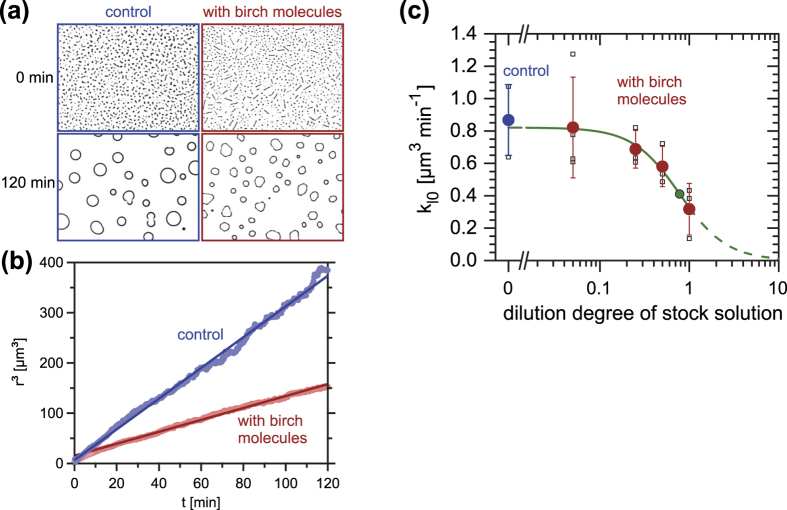
Ice recrystallization inhibition activity of birch pollen molecules. (**a**) Images of polycrystalline ice films after 0 and 120 min of annealing at −8 °C: pure sucrose solution (control; blue) and sucrose solution containing birch pollen #B molecules (red). (**b**) Analysis of the experiments from (**a**) showing the cubic mean ice crystal radius as a function of annealing time (light-shaded circles) and linear fits according to LSW theory (solid lines). (**c**) Rate constant of ice recrystallization *k*_l0_, obtained from LSW analysis, as a function of dilution degree *dd* of the original stock solution (*dd* = 1) containing the birch pollen molecules. Large solid circles represent the average of 3–4 individual measurements (small open squares) at each dilution degree (red) and of a control solution containing no pollen molecules (*dd* = 0; blue). The green line is a sigmoidal fit to the data with an inflection point (green circle) at *dd* ≈ 0.78.

We have performed ice recrystallization experiments using the IRRINA assay^47^,^48^. In IRRINA (Ice Recrystallization Rate INhibition Analysis), a drop of a solution containing antifreeze agents is placed between two microscope glass cover slides forming an approximately 20 μm thick film. This film is rapidly cooled to −50 °C and then warmed to −8 °C. The resulting polycrystalline ice/aqueous solution slurry is shown in Fig. 4a for a pure 45 wt% sucrose solution (control; blue) and for a sucrose solution containing in addition molecules contained in IAP birch pollen washing water (with birch molecules; red). These samples are stored at −8 °C under a microscope and images of the ice crystal size distribution are obtained every 15 s. In the control sample, Ostwald ripening resulted in only a few large round ice crystals remaining after 2 hours, while most of the smaller crystals had melted, Fig. 4a. Note that the total ice volume fraction remains practically constant throughout the entire experiment. In the solution containing the birch molecules, however, a larger number of smaller, hexagonally-shaped ice crystals remained suggesting that the birch molecules have an inhibiting effect on ice recrystallization. This ability can be shown more clearly by examining with image analysis the average radius of all crystals contained in each image as a function of time (Fig. 4b). We plot the cubic mean radius r^3^ as a function of time, resulting in a linear increase of r^3^ with time, just as expected for an Ostwald-ripening process according to Lifshitz-Slyozov-Wagner (LSW) theory^47^,^48^. The rate of the temporal increase in r^3^ can be analysed from linear fitting (solid lines in Fig. 4b) and, after correcting for the experimentally observed ice volume fraction, the corresponding rate coefficient at zero ice volume fraction *k*_l0_ is obtained as a function of the dilution degree of the stock solution *dd* = c(solution)/c(stock solution), see Fig. 4c. Clearly, the value of *k*_l0_ is large in the control solution (blue circle; *dd* = 0), while it becomes smaller with increasing birch pollen molecule concentration, i.e. for larger *dd* values. This behaviour is equivalent to that of antifreeze (glyco)proteins previously investigated with IRRINA^48^. The inflection point of a sigmoidal fit to the data (green line) is defined as the concentration *c*_i_ representing the efficacy of a particular ice recrystallization inhibition agent^48^. The inflection point (green circle) is obtained at a dilution degree of *dd* = 0.78, but the corresponding concentration *c*_i_ cannot be calculated easily, as we do not know the concentration of ice-binding molecules in the stock solution. We did, however, determine that this stock solution contains ~1.1 nmol L^−1^ of ice-nucleating molecules, from which we can infer an upper limit of *c*_i_, see related discussion in Supplementary Note, end of section S.1.1.

The experimental data presented above reveal that molecules released from birch pollen show ice-binding properties similar to antifreeze (glyco)proteins. In fact, the investigated samples exhibit at least three of the four hallmarks of ice-binding activity known from antifreeze (glyco)proteins: (i) they can be purified from multicomponent solutions by binding to slowly growing macroscopic ice; (ii) they induce a characteristic shaping of individual ice crystals; and (iii) they reduce the rate of ice recrystallization in polycrystalline ice samples. In preliminary thermal hysteresis measurements using DSC and cryo-microscopy similar to previously reported protocols^49^,^50^ we did not observe a thermal hysteresis beyond our current detection limit of ~0.2 °C at the IAP stock solution concentration of ice-binding molecules, while at higher concentration the solution became too viscous for such experiments.

### Chemical analysis of birch pollen molecules

**Figure 5:**
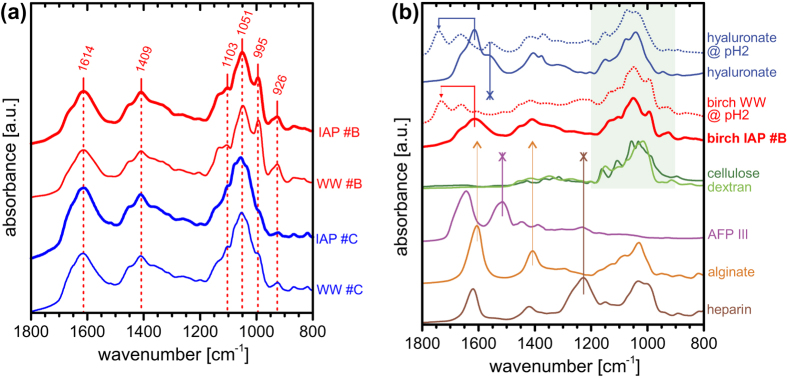
FTIR spectra of dried birch pollen molecules and various reference substances. (**a**) Spectra of ice-affinity purified samples (IAP; thick lines) and of original washing water (WW; thin lines) from two different birch pollen batches #B (red) and #C (blue). Wavenumbers of peak maxima in the IAP #B spectrum are annotated. (**b**) Spectrum of the purified birch pollen #B sample from panel (**a**) (red; birch IAP #B) and reference spectra including those of polysaccharides (green), a protein (antifreeze protein AFP III; magenta), and several functionalized polysaccharides, for details see text. A match of an IR band from reference compounds with that of birch IAP is indicated by an arrow, a mismatch by an X.

**Figure 6:**
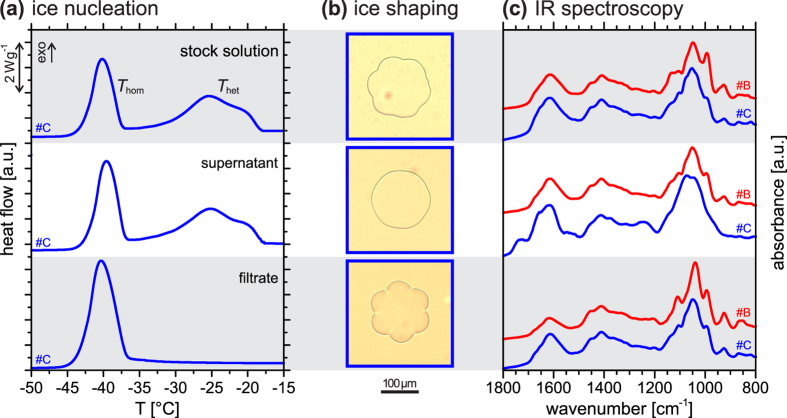
Results obtained with birch washing water #C before and after separation by a 100 kDa cut-off centrifugal filter. (**a**) DSC thermograms show the loss of ice nucleation activity in the filtrate sample (bottom panel), while the supernatant (middle panel) shows the same ice nucleation activity as the stock solution (top panel). (**b**) Ice-shaping experiments show contrary behaviour: the filtrate (bottom panel) shows enhanced ice shaping when compared to the stock solution (top panel); in the supernatant (middle panel) ice shaping is hardly significant. (**c**) IR spectra of washing water #C (blue) and #B (red) before and after centrifugation. The supernatant spectra (middle panel) differ in the polysaccharide characteristic region and in other additional features, while the filtrate spectra (bottom panel) are very similar.

We have investigated the birch pollen molecules using Fourier Transform Infrared (FTIR) spectroscopy in attenuated total reflection mode (see Methods). The spectra in Fig. 5a of dried washing water of birch pollen before (WW; thin lines) and after ice affinity purification (IAP; thick lines) are very similar, suggesting that the ice-binding molecules are the main molecular component of the washing water for both pollen batches #B and #C (red and blue, respectively), see also a more detailed analysis presented in the discussion of Fig. 6c below. The spectra of the two batches differ only slightly in the region around 1100 cm^−1^, see discussion below, and they are similar to that of birch pollen washing water reported in the literature, which was, however, not assigned^51^.

In order to gain more information on the chemical nature of the birch pollen molecules, we analysed the spectrum of an ice-affinity purified birch sample (solid red line in Fig. 5b, birch IAP #B, identical to IAP #B from Fig. 5a). The comparison with spectra of a number of reference compounds (see Supplementary Table S1 for their chemical structure) illustrates several points that we consider to be of particular interest. First, the strong bands in the spectral range from 1200 cm^−1^ to 900 cm^−1^ are characteristic of saccharide moieties^52^,^53^, see comparison to other polysaccharides such as cellulose and dextran (green). Second, the birch spectrum does not contain any pronounced amide I and amide II bands at about 1620–1695 cm^−1^ and about 1520–1590 cm^−1^, respectively, see magenta spectrum of antifreeze protein AFP III for comparison^54^. This absence implies a non-proteinaceous nature of the molecules, in agreement with a previous proposal regarding birch INM^38^,^51^. Third, the birch spectrum also shows strong bands at 1614 cm^−1^ and 1409 cm^−1^, which are characteristic for the asymmetric and symmetric stretches of carboxylates, respectively. The bands are slightly upshifted from ~1550 cm^−1^ and ~1400 cm^−1^ by attachment to saccharide moieties^55^, see the spectrum of dried sodium alginate (orange) for comparison.

A common group of natural acidic polysaccharides are glycosaminoglycans, which consist of disaccharide chains of an amino sugar and an acidic sugar. In Fig. 5b we show spectra of examples of two different classes of glycosaminoglycans: non-sulphated glycosaminoglycans such as hyaluronate (blue) and sulphated glycosaminoglycans such as heparin (brown). Clearly, the birch molecules are not significantly sulphated as their spectrum lacks strong sulphate bands such as those exhibited by heparin at around 1227 cm^−1^ (brown)^56^. Moreover, the birch molecules do not contain amino sugars in significant amounts either as the spectrum lacks the amide band at ~1560 cm^−1^, which is present in the hyaluronate spectrum (blue).

While these comparisons suggest that the birch molecules do not belong to the class of glycosaminoglycans, we can use the latter spectra to support the fact that they contain acidic sugars. When birch washing water is acidified with dilute aqueous HCl to pH2 before drying (red solid and dotted spectra, respectively), the carboxylate bands at 1614 cm^−1^ and 1409 cm^−1^ are clearly reduced in intensity in favour of a band at 1741 cm^−1^, characteristic for protonated carboxylic acids (red arrow). The remaining band at ~1660 cm^−1^ may be attributed to residual water in the sample^55^. A nearly identical behaviour (blue arrow) is observed when a solution of sodium hyaluronate is acidified to hyaluronic acid (blue dotted), supporting the presence of acidic sugars in birch pollen molecules. In contrast to hyaluronic acid, acidification of the birch molecules did not reveal any pronounced band at ~1560 cm^−1^, further pointing to the absence of significant amounts of amide moieties.

In summary, the analysis of the IR spectra shown in Fig. 5b reveals that the dominant components of the birch pollen molecules are non-sulphated polysaccharides containing a significant number of carboxylic acid moieties. Hence at neutral pH conditions these molecules may be considered as anionic polysaccharides. Our analysis is in agreement with previous findings which inferred a non-proteinaceous nature of birch pollen INM based on experiments that did not reveal any loss of ice nucleation activity after either heating to 172 °C for one hour, or addition of 6 mol L^−1^ guanidinium chloride, or treatment with the enzymes pronase E, papain, and trypsin^26^,^38^. Instead, it was suggested previously that the INM may be polysaccharides^26^,^38^.

We performed additional experiments as an independent evidence that the INM are anionic polysaccharides. It is well known that solutions of alginate form gels when Ca^2 + ^(aq) ions are added: the monovalent carboxylate groups form complexes with the bivalent Ca^2 + ^(aq) ions, thereby linking different polysaccharide molecules to form a macroscopic gel^57^,^58^. Indeed, addition of Ca^2 + ^(aq) to ice affinity-purified birch #C washing water resulted in flocculation, similar to alginate (see Supplementary Fig. S3 and discussion). These experiments thus support the conclusions from the FTIR measurements above that the molecules released by birch pollen are anionic polysaccharides.

### Similarities and differences between ice-nucleating and ice-binding molecules

The ice affinity-purified sample contains both ice-nucleating as well as ice-binding molecules. Therefore, the question arises whether the two functions are caused by the same molecule or different types of molecules. In order to characterize the homogeneity of the molecules as well as their approximate molecular size we have performed experiments using centrifugal filtration.

We separated molecules larger than 100 kDa from smaller molecules by centrifuging the sample containing both ice-nucleating molecules and ice-binding molecules through a centrifugal filter with a 100 kDa molecular weight cut-off to investigate a potential size dependence of the ice-binding and ice nucleation activity. We used this cut-off size because experiments by Pummer *et al*. revealed that the ice-nucleating molecules passed a 300 kDa cut-off filter, while ice nucleation activity was lost in the filtrate of a 100 kDa cut-off filter^26^,^38^.

Here we performed emulsion ice nucleation measurements with the stock solution of birch #C before centrifugal filtration, as well as with the supernatant and with the filtrate after centrifugal filtration, see Methods. The resulting thermograms are shown in Fig. 6a. The supernatant (middle panel) exhibits the same ice nucleation activity as the stock solution (top panel), even after several washing cycles. In contrast, the filtrate did not show any heterogeneous ice nucleation signal indicating the absence of capable ice nucleators, in agreement with previous studies^26^,^38^. The same result was obtained studying a birch #B sample (see Supplementary Fig. S4). In addition, we investigated the ice-shaping ability of the three samples. In the stock solution ice crystals grew into a flower-like shape (top panel in Fig. 6b). The supernatant did not show significant ice shaping (middle panel), while the filtrate exhibits more pronounced ice crystal shaping (bottom panel) than is observed in the stock solution. Again, experiments with birch #B are consistent with these results (see Supplementary Fig. S4).

Finally, we obtained infrared spectra of the stock solution, supernatant and filtrate for both birch batches #B and #C (Fig. 6c). Clearly, the spectra of both the supernatant and the filtrate reveal the dominance of polysaccharides bearing carboxylic moieties (compare Fig. 5b). All spectra show a striking number of similarities suggesting that these samples are dominated by a few species only. We analysed the samples independently by fluorophore-assisted carbohydrate electrophoresis, which verified this conclusion (see Supplementary Fig. S5 and related discussion). Furthermore, the infrared spectra of the filtrate are notedly structured, and the #B filtrate spectrum is particularly well resolved, which may indicate that it contains only a single species of ice-binding polysaccharide with a comparatively lower content of carboxylate groups. Only the #C supernatant spectrum shows rather different patterns in the polysaccharide region between 900 and 1200 cm^−1^ and features additional bands at about 1242 cm^−1^, 1530 cm^−1^, and 1737 cm^−1^. We know from the ice nucleation experiments that birch #C contains two ice nucleators (top and middle panel in Fig. 6a), whereas birch #B contains only one nucleator with an onset temperature equivalent to that of the more active ice nucleator in #C (Fig. 1). Accordingly, the additional features in the #C supernatant spectrum may be attributed to the less active ice nucleator, which is present in significant amounts only in the #C supernatant. Finally, we use the spectra of birch #C to estimate the relative amounts of ice-nucleating and ice-binding molecules by using a linear combination of the spectra of supernatant and filtrate to reconstruct the stock solution spectrum. This analysis revealed that the supernatant spectrum cannot contribute more than ~11% to the stock solution spectrum. Given that this is a crude upper limit only and that the ice-nucleating molecules have a larger molecular weight than the ice-binding molecules, the molar ratio of ice-nucleating to ice-binding polysaccharides is likely much smaller than 0.1. We could not determine the molecular weight of the ice-binding molecules. But preliminary experiments using smaller cut-off filters indicate that they may be larger than several kDa, which coincides with the molecular weight of the smallest natural antifreeze (glyco)proteins (~3 kDa) as well as with the threshold value (~1 kDa) for ice recrystallization inhibition activity of synthesized poly(vinyl alcohol) polymers of well-defined molecular weight^59^.

The importance of the hydroxyl functionalities of the polysaccharides for both ice-nucleating and ice-binding activity is supported by the fact that the addition of borate, which can form esters with polysaccharide hydroxyl groups, diminishes both functions, i.e. heterogeneous ice nucleation and ice shaping (see Supplementary Fig. S6). Moreover, acidification by HCl to pH2 in order to protonate the carboxylate functions, see Fig. 5b, does not affect the onset temperature for ice nucleation nor ice shaping (see Supplementary Fig. S6), suggesting that the carboxylate functions are not directly involved in the interaction with ice. We note, however, that HCl addition does reduce the magnitude of the ice nucleation signal, and the original signal magnitude is not recovered after re-neutralization with NaOH. This behaviour indicates that some ice nucleators are lost irreversibly after HCl addition, which may be indicative of acid-catalysed sugar hydrolysis of the ice-nucleating polysaccharides.

In summary, these observations suggest that the birch pollen washing water contains both ice-nucleating polysaccharides, larger than ~100 kDa, that do not bear significant ice-shaping activity as well as ice-binding polysaccharides, significantly smaller than ~100 kDa, that do not show ice-nucleating activity. Thus, the ice-nucleating polysaccharides are larger than the ice-binding ‘antifreeze’ polysaccharides, which coincides with the equivalent consideration that ice-nucleating proteins are typically much larger than antifreeze proteins^3^,^32^,^33^,^34^,^35^. Ice nucleation and ice-binding both require that the chemical moieties of the molecules allow for an interaction with ice-like ordered water molecules, but ice nucleation additionally requires that the active site of the molecules is large enough to support the formation of a critical ice embryo.

### Other pollen types

**Figure 7:**
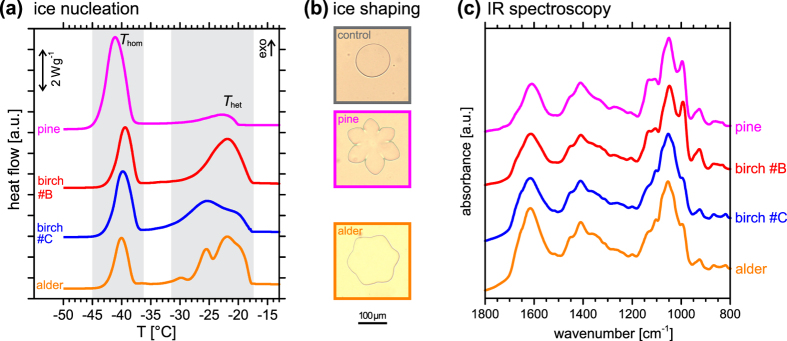
Comparison of washing water from pine and alder pollen with that of birch pollen batches #B and #C. (**a**) Pine pollen WW shows one heterogeneous ice nucleation signal (magenta), similar to birch #B WW (red), and alder pollen WW shows several heterogeneous ice nucleation signals (orange) similar to birch #C WW (blue). (**b**) Pine and alder pollen WW show ice shaping. (**c**) FTIR spectra of dried residues of pine and alder pollen WW (magenta and orange, respectively) in comparison to birch pollen #B and #C WW (red and blue). We note the close similarity of the pine and birch #B spectra and those of alder and birch #C, suggesting that they contain very similar anionic polysaccharides, with similar ice nucleation behaviour, see panel (**a**).

It has been reported previously that washing water of several kinds of pollen induce heterogeneous ice nucleation^26^. Hence, we wondered whether ice-nucleating molecules released from other pollen types may also show ice-binding properties. To answer this question, we investigated pollen from European alder (*Alnus glutinosa*) and Scots pine (*Pinus sylvestris*). These pollen are known to be good ice nucleators^26^,^60^,^61^, as confirmed by the ice nucleation signals in DSC experiments with dilute solutions of washing water from alder and pine pollen (orange and magenta in Fig. 7a). The samples exhibit heterogeneous ice nucleation signals at temperatures of up to −17 °C and −20 °C, in agreement with previous ice nucleation studies of such pollen suspended in microliter or picoliter droplets^7^,^12^,^26^,^38^,^39^,^60^,^61^. More importantly, the washing water of pine pollen and of alder pollen also show ice crystal shaping (Fig. 7b), indicating that these pollen both contain ice-binding molecules similar to birch pollen. These observations suggest that ice-binding may be a general behaviour of molecules released from pollen that also contain ice-nucleating molecules. We also performed FTIR experiments on pine and alder pollen washing water residues, which we compare to those of birch pollen in Fig. 7c. Clearly, the spectra of pine (magenta) and alder (orange) reveal the presence of polysaccharides bearing carboxylic moieties, just as for birch (red and blue). Moreover, the spectrum of pine is practically identical to that of birch #B, and that of alder to that of birch #C. This observation is consistent with the number of ice nucleators observed in Fig. 7a in the respective pollen washing water: only one nucleator in pine and birch #B, and two or more in birch #C and alder.

## Discussion

Polysaccharides constitute a common class of molecules that interact with ice in nature either by triggering ice nucleation or by inhibition of ice nucleation and growth. For example, ice nucleators found in the Afro-alpine plant *Lobelia telekii* are supposedly carbohydrates^21^. In contrast, a high molecular weight polysaccharide from *Bacillus thuringiensis* was found to inhibit ice nucleation^62^. Finally, another polysaccharide with a fatty acid component from the Alaskan beetle *Upis ceramboides* exhibited a pronounced thermal hysteresis due to ice binding^29^. The birch pollen polysaccharides studied here add to this group of ice-active saccharides. From FTIR spectroscopy we identified two slightly different polysaccharides both bearing carboxylic moieties. They appear to have a very similar chemical structure, the main difference appears to be their size: one larger than 100 kDa and responsible for ice-nucleation activity, the other significantly below 100 kD and exhibiting only ice-binding properties. The size requirement for ice-nucleating activity observed here is in agreement with the recent finding that the correlation between size and ice nucleation temperature of molecular ice nucleators corresponds to that of the temperature dependence of the critical ice embryo size obtained from classical nucleation theory^38^. We note that the ice-nucleating molecules can pass the ice affinity purification procedure and also show minute ice shaping activity. The IR spectrum of the supernatant of birch sample #B closely resembles that of the filtrate of sample #B and of #C. This similarity suggests that the ice-binding molecules may be fragments of the larger ice-nucleating molecules or, alternatively, that the ice-nucleating molecules are in fact adducts or clusters of several (at least two) smaller ice-binding molecules.

It seems, therefore, reasonable to propose that the active site of the ice-nucleating molecules and the ice-binding site of the ice growth-inhibiting molecules may not be distinct, but in fact may originate from the same molecular moieties. Our observations do not contradict but indeed support the consideration that the same type of molecule can show both ice-nucleating as well as ice-binding ability, in agreement with the notion recently discussed by Davies that apart from the size of their active site there is no principal difference in the mechanistic interaction of antifreeze or ice-nucleating proteins with ice^3^. The fact that the active site of ice-nucleating proteins is usually larger than that of antifreeze proteins may explain why the latter are usually inapt or poor ice nucleators.

It has not escaped our notice that the two seemingly opposing properties of pollen polysaccharides opens the question of their primary biological function: is it the ability of ice-nucleating polysaccharides to trigger precipitation in the atmosphere, or is it the antifreeze protection by ice-binding polysaccharides during periods of night frost in early springtime, or are both functions important by triggering extracellular ice nucleation and inhibiting intracellular ice growth? Antifreeze protection by extracellular ice nucleation is supported by a correlation between the ice nucleation ability of pollen species and the time of year they are released^60^,^61^. The smaller ice-binding polysaccharides described above may add to this protection mechanism. To answer this question requires more thorough biological studies and may involve a comparison to pollen types that do not contain molecules with both types of functions.

Furthermore, it is certainly notable that, to the best of our knowledge, the pollen polysaccharides are the first known ice-binding molecules that can safely be assumed to be present in the atmosphere. Although we consider it to be relatively unlikely that ice-binding polysaccharides from pollen will significantly affect the shape of atmospheric ice crystals, ice-nucleating polysaccharides from pollen may affect atmospheric ice nucleation. While the atmospheric concentration of entire pollen particles is comparably low due to their large size and corresponding settling velocities^63^, the water-soluble polysaccharides may be transferred into the atmosphere even without their pollen particle host: a recent study proposed a bubble-bursting process that transfers soil organic matter into the atmosphere by ejecting small aerosol particles^16^. Hence, it appears at least conceivable that the polysaccharides are released from their pollen particles during rain^64^, droplets of which then also trigger bubble bursting and ejection of particles containing the released molecules^16^. Such a process would be in agreement with observations of enhanced concentrations of biological and ice-nucleating particles during and after rainfall^14^.

## Methods

### Materials

Pollen were obtained from GREER as non-defatted samples: European white birch (*Betula pendula*; item#: RM527, LOT#: P1329870-1/-2, P3317516-1, and P1329872-1 termed here batch #A, batch #B, and batch #C, respectively. #A was collected on March 30, 2011 in France, contained 2.16% plant parts, and 0.39% contamination by spores or other pollen. #B was collected on April 18, 2013 in France, contained 3.04% plant parts, and no contamination. #C was collected in March, 2011 in France, contained 0.98% plant parts, and 0.29% contamination by spores or other pollen.), European alder (*Alnus glutinosa*; item#: RM523, LOT#: P125246) and Scots pine (*Pinus sylvestris*; item#: RM464, LOT#: 100PP464-5(1)). Bi-distilled water was prepared freshly each day, all other chemicals were obtained commercially: sucrose (AppliChem Panreac, 99%); glucose (Sigma Aldrich, 95%); NaCl (AnaLaR NORMAPUR/VWR, 100%); Span 65 (sorbitan tristearate; Merck); heparin sodium salt (Sigma Aldrich); hyaluronic acid sodium salt (Sigma Aldrich); dextran (Sigma Aldrich, 150 kDa); alginic acid sodium salt (Alfa Aesar); microcrystalline cellulose (Sigma Aldrich); natural type 3 antifreeze protein, AFP III, from polar fish of suborder *Zoarcoidei* (A/F-Protein); methylcyclohexane (Acros Organics, 95%) and methylcyclopentane (Acros Organics, 99%); Na_2_B_4_O_7_·10H_2_O, HCl (0.1 M, VWR Chemicals) and NaOH (0.1 M, VWR Chemicals).

### Sample preparation

Pollen suspensions for DSC or BINARY freezing experiments were prepared by suspending several mg of pollen grains in a few mL of freshly bi-distilled water (e. g., 40 mg pollen were suspended in 10 mL water) by manual shaking in a test tube for a few minutes. Pollen washing water for DSC or BINARY freezing experiments was prepared by filtration of the pollen suspension through a 0.2 μm polyethersulfone syringe filter (VWR). Pollen washing water for ice affinity purification required large amounts of pollen material. Several aliquots of a total of about 100 g of birch pollen (batches #B or #C) were suspended consecutively in a total of about 500 mL freshly bi-distilled water (e.g., 15 g in 100 mL water). After each suspension procedure (15–60 minutes of stirring with a magnetic stirrer) the pollen grains were filtered out of the suspension through a 5 μm cellulose nitrate membrane filter (Sartorius) and washed with water several times. The resulting raw filtrate was used for ice affinity purification. Normally, pollen suspensions and washing water were prepared freshly on the day of measurement, but when necessary filtrated washing water and ice affinity purified washing water samples were stored in a freezer (at −18 °C) to prevent degradation or aging and were used within a few weeks. We did not observe any significant inactivation during that time.

### Differential Scanning Calorimetry (DSC)

Ice nucleation behaviour of pollen washing water in inverse (water-in-oil) emulsions was investigated with a differential scanning calorimeter (TA Instruments Q100). Exothermic processes such as homogeneous or heterogeneous ice nucleation were measured as the heat flow difference between the sample pan and an empty reference pan. The calorimeter has been calibrated exhaustively as a function of temperature and cooling/heating rate^40^,^65^. Ice nucleation temperatures were investigated at a cooling rate of −5 °C min^−1^ and ice melting temperatures usually at a heating rate of +1 °C min^−1^ (except for NaCl solutions, which were studied at +5 °C min^−1^). Ice nucleation temperatures (*T*_hom_ and *T*_het_) were determined as the onset of the corresponding exothermic signal, and ice melting temperature (*T*_m_) as the inflection point of the corresponding endothermic signal. The inverse emulsions were prepared by mixing 2 mL of an organic phase, consisting of 7 wt% of the surfactant Span 65 dissolved in a 1:1 by volume mixture of methylcyclohexane and methylcyclopentane, and 2 mL of the aqueous phase of interest. This mixture was stirred with a high-speed disperser (IKA Ultra-Turrax T25 basic) at room temperature for 10 min at 20,000 rpm to generate an emulsion containing aqueous droplets about 1–10 μm in size. About 7 mg of an emulsion was transferred into a hermetically-sealed aluminium DSC pan for immediate measurement.

### Optical freezing array (BINARY)

To investigate the freezing behaviour of both pollen suspensions and washing water over a broad concentration range an optical freezing array (BINARY: Bielefeld Ice Nucleation ARraY) was used^66^. Droplets of 1 μL volume were positioned on a hydrophobic glass slide in a 6 × 6 array. The droplets were separated from each other using a polydimethylsiloxane spacer to prevent a water vapour transfer from unfrozen to frozen droplets during experiments. The droplets were cooled at a rate of −1 °C min^−1^ until all droplets were frozen. Freezing of the droplets was detected by the associated change in droplet transparency using a digital camera (QImaging MicroPublisher 5.0 RTV).

### Ice affinity purification

The ice-binding molecules contained in the raw birch pollen filtrate washing water were purified using the method of ice affinity purification (IAP)^43^. After two IAP cycles the resulting purified washing water was filtered through a 0.2 μm polyethersulfone syringe filter (VWR) and the contained molecules were concentrated by removing some of the water under reduced pressure at a temperature of 20–30 °C.

### Ice-shaping experiments

Different types of samples were used in the ice-shaping experiments. For birch pollen, raw washing water after filtration and concentrating at 90 °C for 3 hours at ambient pressure was used as well as solutions obtained after ice affinity purification, see above. (We note that the original purpose of heating to 90 °C for several hours was the corroboration that the ice-shaping molecules are not heat sensitive, suggesting that they are non-proteinaceous; however, elevated temperatures are not required for concentrating, which can also be performed at room temperature and reduced pressure, see the treatment of birch pollen above, and alder and pine pollen below.) The birch pollen molecules were analysed in a 30 wt% aqueous sucrose solution, and a pure 30 wt% aqueous sucrose solution was also analysed as a control. For alder and pine pollen the raw washing water after filtration and concentrating at room temperature and reduced pressure was used without further treatment. 5 μL of a sample solution was embedded as a thin film between two glass coverslips (14 mm diameter) and sealed with silicone grease. This sample cell was then placed into a temperature-controlled cryostage (Linkam, BCS196) attached to an optical microscope (Olympus BX51) equipped with a digital camera (QImaging MicroPublisher 5.0 RTV). First, the sample was rapidly cooled to −30 °C (for purified samples) or −40 °C (for raw filtrate samples) to form polycrystalline ice within the film, and subsequently the sample was slowly heated to a temperature just below the melting point until only a few ice crystals remained. (The absolute temperature differed between samples due to differences in solute concentrations.) In further cooling and heating cycles at very small cooling and warming rates of ±0.1 or ±0.01 °C min^−1^ most of these crystals were melted until finally only one or two ice crystals were obtained in the field of view. The very slow growth of these crystals was observed at a very small cooling rate of −0.01 °C min^−1^ with digital microphotographs taken every 6 s, see previous papers for more details^47^,^48^,^67^.

### Ice recrystallization inhibition

The kinetics of ice recrystallization in aqueous 45 wt% sucrose solutions containing birch pollen molecules was studied using the Ice Recrystallization Rate INhibition Analysis (IRRINA) assay^47^,^48^. The birch pollen samples were used after ice affinity purification and investigated in a dilution series. The INM concentration of the stock solution was determined from BINARY freezing measurements, see Supplementary Fig. S1 and related text. A thin sample film is formed by pressing 2 μL of sample solution between two glass coverslips (14 mm diameter). This sample cell is placed on the same cryostage/optical microscope setup as described in the previous paragraph (ice-shaping experiments). Initially, the sample was cooled at a rate of −20 °C min^−1^ to −50 °C in order to produce a polycrystalline ice film. After heating to −8 °C the sample temperature was held constant for 2 hours while microphotographs were taken every 15 s. The size of all ice crystals in the field-of-view was obtained using image analysis and the corresponding cubic mean radius was analysed as a function of time using LSW theory; for detail see previous publications^47^,^48^.

### Infrared spectroscopy

Fourier transform infrared (FTIR) spectroscopy was performed in attenuated total reflection (ATR) mode on one of two FTIR spectrometers (Bruker IFS 66 v and IFS 66 s) equipped with an ATR unit containing a diamond reflection element with nine active reflections (DuraSampleIRII, Smiths) and a mercury cadmium telluride detector as described previously^68^. 20–40 μL of a sample solution were pipetted onto the ATR crystal and dried at room temperature under a stream of dry air for at least 10 min. Thereafter, the spectra of the dry sample films were recorded with a spectral resolution of 2 cm^−1^ using either 1024 scans (IFS 66 s) or 512 scans (IFS 66 v). Absorbance values were divided by the corresponding wavelength to correct for the wavelength dependence of the penetration depth of the evanescent wave.

### Centrifugal filtration

Centrifugal filters with a 100 kDa molecular weight cut-off (Vivaspin 500, MWCO 100, Sartorius) were used to separate molecules of different size present in the birch pollen washing water. Initially, the stock solution was centrifuged for about 30–60 min (depending on the initial volume) at 5,000 rpm, the supernatant was shaken occasionally, about every 5–10 min. Thereafter, the filtrate was removed from the tube and stored separately. Then several aliquots of pure water (~300–500 μl each) were added and centrifuged subsequently (about 7 times, each for 5–10 min) to wash off any residual smaller molecules from the supernatant. Afterwards the remaining supernatant was diluted with water up to the initial volume. The entire procedure was performed independently with three stock solutions: with washing water of birch #B and #C, and with a concentrated, twice ice affinity-purified sample of birch #B.

### Data availability

Figure source data of the x-y-plots shown in this paper have been deposited in the public institutional repository (PUB) of Bielefeld University (doi: 10.4119/unibi/2907691).

## Additional Information

**How to cite this article**: Dreischmeier, K. *et al*. Boreal pollen contain ice-nucleating as well as ice-binding ‘antifreeze’ polysaccharides. *Sci. Rep.* **7**, 41890; doi: 10.1038/srep41890 (2017).

**Publisher's note:** Springer Nature remains neutral with regard to jurisdictional claims in published maps and institutional affiliations.

## Supplementary Material

Supplementary Information

Supplementary Video S1
