# Supplementary material for: Boreal pollen contain ice-nucleating as well as ice-binding ‘antifreeze’ polysaccharides
Source: Sci Rep. 2017 Feb 3;7:41890. doi: 10.1038/srep41890 (PMC5291224; doi:10.1038/srep41890)
Supplement: Supplementary Information [file srep41890-s1.pdf]

# Supplementary Information

## Boreal pollen contain ice-nucleating as well as ice-binding ‘antifreeze’ polysaccharides

Katharina Dreischmeier[1], Carsten Budke[1], Lars Wiehemeier[1,2], Tilman Kottke[2], Thomas Koop[1,3]

[1] Bielefeld University, Faculty of Chemistry, Atmospheric and Physical Chemistry, D-33615 Bielefeld, Germany

[2] Bielefeld University, Faculty of Chemistry, Physical and Biophysical Chemistry, D-33615 Bielefeld, Germany

[3] Bielefeld University, Center for Molecular Materials, D-33615 Bielefeld, Germany

Correspondence and requests for materials should be addressed to T. Koop
(Email: thomas.koop@uni-bielefeld.de)

**S1. Supplementary Note**

**S1.1. Concentration of ice-binding molecules in ice-affinity purified birch pollen washing water.** For the ice recrystallization inhibition experiments with the IRRINA assay (see Methods) knowledge of the concentration of ice-binding molecules in the aqueous solution after ice affinity purification is desirable. We estimated a lower limit of this concentration by first determining the number of ice-nucleating molecules in the stock solution. For this purpose, we performed freezing experiments with the BINARY setup (see Methods). Supplementary Figure S1 shows the cumulative number of ice nucleators per pollen grain, $n_n(T)$, contained in aqueous birch pollen suspensions and their corresponding washing water as a function of temperature. Freezing experiments with serially diluted solution droplets were performed. Using the droplet freezing temperatures, $n_n(T)$ can be obtained from the known mass concentration of pollen using a value of 24 µm as the birch pollen grain diameter and a value of 1 g $cm^{-3}$ as the dry pollen grain density.[1] Supplementary Figure S1 shows that the birch pollen suspension as well as the washing water contains about $10^4$ INM per pollen grain independently of the pollen batch used (#A or #B), in agreement with previous independent determinations of $n_n(T)$.[2,3]

In the next step, we determined $n_n(T)$ in the stock solution after ice affinity purification by performing freezing experiments on serially diluted solution droplets with the BINARY setup as described above. Different $n_n(T)$ data sets were calculated by assuming different values for the unknown stock solution INM concentration. That concentration whose $n_n(T)$ data matches best with a fit of the $n_n(T)$ of the birch pollen washing water of known concentration from Supplementary Fig. S1 at temperatures from −17 to −25 °C was determined by minimizing the deviation between the data and the fit. As a result we obtained an INM concentration after ice affinity purification that corresponds to that of washing water of about 700 ±200 mg pollen grains per mL of water, i.e., about 1.6 ±0.2 nmol $L^{-1}$ of ice-nucleating molecules. That solution was mixed with a concentrated aqueous sucrose solution, yielding a final stock solution for the IRRINA experiments that contained 45 wt% of sucrose and ~1.1 ±0.2 nmol $L^{-1}$ of ice-nucleating molecules (dilution degree of *dd* = 1). This stock solution was then investigated at different dilution degrees in the IRRINA experiments shown in Fig. 4 of the main article. We obtained a value for the inflection point in Fig. 4c of *dd* = 0.78 ±0.1, i.e. that solution contained ~0.8 ±0.2 nmol $L^{-1}$ ice-nucleating molecules. However, the concentration of ice-binding molecules responsible for ice recrystallization inhibition is not known, but we can estimate a lower limit for their concentration as follows. The FTIR experiments shown in Fig. 6c of the main article indicate that the molar ratio of ice-nucleating molecules to ice-binding molecules is smaller than

about 0.11. Hence, we can infer a lower limit for the ice recrystallization inhibition efficacy concentration of $c_i \geq 7 \pm 2$ nmol $L^{-1}$ of ice-binding molecules.

**S1.2. Ice nucleation in aqueous solutions containing birch pollen INM.** We studied the effect of additional solutes on the activity of birch pollen INM. For this purpose, we added quantified amounts of either the electrolyte NaCl, the hydrophilic organic molecule glucose, or a NaCl/glucose mixture to the birch pollen washing water. These aqueous solutions were then prepared into inverse emulsions and were studied using DSC. The results for aqueous solutions containing 2.5 wt%, 7.5 wt% and 12.5 wt% NaCl are shown in Supplementary Fig. S2a as the green, blue and orange lines, respectively. $T_{hom}$ as well as $T_{het1}$ and $T_{het2}$ decrease with increasing NaCl concentration similar to previous studies with other ice nucleators.[4–6] These data, together with results from aqueous glucose and aqueous NaCl/glucose solutions, are shown in Supplementary Fig. S2b as a function of the solute mass fraction. The same data are shown in Supplementary Fig. S2c as a function of the total solute molality $b_{tot}$, i.e., assuming NaCl to be fully dissociated in the solutions ($b_{tot} = b_{gluc}$, $b_{tot} = 2 \cdot b_{NaCl}$, or $b_{tot} = b_{gluc} + 2 \cdot b_{NaCl}$). The data points of the different solute types shown in Supplementary Figs. S2b and S2c deviate significantly from each other, e.g., by up to ~20 °C at a solute mass fraction of 0.2 and by up to ~15 °C at a total solute molality of 5.5 mol $kg^{-1}$. Therefore, the data from Supplementary Figs. S2b and S2c were analysed using a water-activity-based ice nucleation approach, which suggests that ice nucleation in different solutions is independent of the type of solute when plotted in terms of the solutions' water activity.[4,7–9] Results of this analysis are shown in Supplementary Fig. S2d, where $T_{hom}$, $T_{het1}$ and $T_{het2}$ as well as the ice melting temperature $T_m$ are plotted as a function of water activity (see next section for details). Clearly, all data collapse to form narrow bands of points, independently of the nature of the solute. This behaviour indicates that the ice-nucleating ability of birch pollen INM is not directly affected by the presence of solutes, i.e., the solutes NaCl and glucose apparently do not lead to significant chemical or structural modification of the ice-nucleating active site.

The three solid lines describing the $T_{hom}$, $T_{het1}$ and $T_{het2}$ data (blue, red and green lines, respectively) in Supplementary Fig. S2d are not polynomial fits to the data. Instead they were constructed by horizontally shifting the thermodynamically defined ice melting point curve (black line, $T_m$) until they match the data.[7] Each horizontal shift in water activity, $\Delta a_w$, is representative of a particular ice nucleation rate coefficient[8] and, for a specific set of experimental conditions (e.g., cooling rate and sample size), it is representative of an experimentally observable $T_{hom}$ or $T_{het}$ curve.[4,7] The corresponding values resulting from the analysis presented in Supplementary Fig. S2d are $\Delta a_{w,hom} = 0.315$, $\Delta a_{w,het1} = 0.159$, and $\Delta a_{w,het2} = 0.195$.

**S1.3. Water activity of aqueous solutions.** In order to apply the water-activity based approach to the homogeneous[10,11] and heterogeneous[4,7,8] ice nucleation data of aqueous solutions containing birch pollen INM, parameterizations for the water activity $a_w$ of aqueous solutions of glucose, NaCl, and NaCl/glucose mixtures were required. For glucose we employed the parameterization of Zobrist et al.,[12] which provides $a_w$ as a function of temperature and glucose mass fraction. It is repeated here in Supplementary Equation (S1):

$$a_w(w,T) = \frac{1 + a\cdot w}{1 + b\cdot w + c\cdot w^2} + (T - T^*)\cdot(d\cdot w + e\cdot w^2 + f\cdot w^3 + g\cdot w^4), \qquad \text{(S1)}$$

where *w* is the solute mass fraction, *T* is temperature in kelvin, $T^*$ = 298.15 K is a reference temperature, and the fitting coefficients *a*-*g* are given in Supplementary Table S2. Supplementary Equation (S1) was also used to fit the water activity of aqueous NaCl solutions with data at different molality (0.5-5.0 mol $kg^{-1}$) and temperature (263.15-293.15 K), taken from the Aerosol Inorganics Model (http://www.aim.env.uea.ac.uk/aim/aim.php),[13–15] see Supplementary Table S2 for parameters.

For the aqueous NaCl/glucose solutions [1:2 mole ratio] no parameterization was available. Therefore, the water activity of the investigated solutions at the experimentally determined ice melting temperature was derived using the parameterization of Koop and Zobrist.[7] This $a_w$ value was then applied to the freezing temperatures of the same solution by assuming that the water activity in these solutions is temperature-independent. This assumption appears reasonable based on the opposing small temperature dependence of $a_w$ in solutions of constant mass fraction of NaCl and glucose, respectively.

**S1.4. Additional characterisation of birch pollen polysaccharides.** In order to confirm the importance of polysaccharide functionalities for both ice-nucleating activity and ice-binding activity of birch pollen molecules, we studied the effect of borate addition on ice nucleation and ice binding. It is known that borate forms ester complexes with sugar hydroxyl moieties.[16,17] Addition of borate ions, $BO_3^{3-}$, to saturation by supplying $Na_2B_4O_7\cdot 10H_2O$ to a diluted IAP2 solution of birch pollen #C washing water resulted in the deactivation of the ice-nucleating molecules, as the heterogeneous ice nucleation signal in DSC experiments of the diluted original solution (purple line in Supplementary Fig. S6a) practically disappears after addition of borate (green line). Only the homogeneous ice nucleation signal remains (steep increase in heat flow below about –38 °C), which is shifted to slightly lower temperature when compared to the diluted original solution (below about –36 °C) due to the colligative melting point depression and corresponding ice nucleation temperature depression upon addition of the solute borate.[7,10] Moreover, also the ice-shaping ability is lost in moderately

concentrated original birch #C solutions after addition of borate, see Supplementary Fig. S6b. A crystal growing in a moderately concentrated solution of ice-binding molecules still showed a minimal hexagonal shape (top image in Supplementary Fig. S6b), while an ice crystal grew into an entirely round shape after addition of borate (bottom image), implying the loss of ice-binding activity. (Note that experiments at higher concentrations were impracticable because borate crystals precipitated, thereby affecting the shape of the ice crystals.) Together, these experiments support the notion that polysaccharide hydroxyl groups are essential for both the ice-nucleating function as well as the ice-binding function, in agreement with independent conjectures of Pummer et al.[18,19]

In another set of experiments we studied the effect of pH on ice nucleation and ice binding. Addition of $H_3O^+$ ions by supplying dilute aqueous HCl to a dilute solution of birch pollen #C washing water had only a relatively small effect on ice nucleation ability (red line in Supplementary Fig. S6c). The heterogeneous ice nucleation onset temperature was not affected when compared to a sample which was diluted with pure water instead of HCl (brown line). However, the peak area of the heterogeneous ice nucleation signal was diminished suggesting a reduction in the total number of active ice-nucleating molecules. In order to determine whether this reduction was reversible or irreversible we performed a second set of experiments. An aliquot of the acidified solution from the first experiment (red) was re-neutralized to pH8 by subsequent addition of dilute aqueous NaOH, resulting in a moderate change in the ice nucleation signal at temperatures below about –28 °C only (blue line in Supplementary Fig. S6c), but not in a full recovery to the original signal (brown line). In addition, re-neutralization did not affect the onset or magnitude of the heterogeneous ice nucleation signal at higher temperatures (between –18 °C and –26 °C). These experiments suggest that the active site for ice nucleation is not directly affected at low pH, from which we infer that the carboxylic moieties are not part of the active site. The irreversible loss of ice-nucleating molecules upon the initial treatment with HCl may be caused by acid-catalysed sugar hydrolysis of the ice-nucleating polysaccharides. Finally, also the ice-shaping activity of the ice-binding polysaccharides is not visibly affected at low pH, as ice crystals grew in a ‘flower-like’ shape in the concentrated original solution (top image in Supplementary Fig. S6d) as well as after treatment with dilute HCl (bottom image). These observations imply that the activity of ice-binding molecules is not affected at low pH, which may suggest that the carboxylic moieties are not part of the ice-binding site.

We also performed experiments using gel electrophoresis in order to characterize the birch pollen polysaccharides further. In contrast to the usual application of Polysaccharide Analysis using Carbohydrate gel Electrophoresis (PACE),[20] we did not focus on the separation of oligomers after hydrolysis of the polysaccharide, but aimed for the original high molecular weight compounds. Such

analysis is challenging, and standard protocols have been developed only recently.[21,22] The separation velocity is dependent on the size of the polymer as well as on the number of charges per molecule, both of which need to be taken into account in the interpretation. In the Fluorophore-Assisted Carbohydrate Electrophoresis (FACE), the separation in the gel is enhanced by using a charged fluorophore as a label.[23] We followed a protocol modified from Goubet et al. (2011) in which the triply charged fluorophore ANTS (8-Aminonaphthalene-1,3,6-trisulfonic acid) is the charge-bearing active component.[21] Originally, an uncharged fluorophore was used (AMAC; 2-Aminoacridone) following a PACE procedure, which, however, was not successful in our experiments, in agreement with previous observations by Tawada et al.[24]

The gel of the #C sample before and after ice-affinity purification (see Supplementary Fig. S5a) indicates the existence of at least three polysaccharide species, of which one is highly abundant. Accordingly, the birch pollen do not release a diverse range of biological compounds, but instead only a few polysaccharides of defined molecular weight and charge-to-size ratio. Judging from the oligomeric maltodextrin standard, the compounds might be classified misleadingly as uncharged low molecular weight oligomers, but such interpretation can be excluded by the results presented in the main article. Therefore, we analysed a 150 kDa dextran after labelling with ANTS and investigated supernatant and filtrate of the birch #C IAP1 after separation by a 100 kDa cut-off filter (see Supplementary Fig. S5b). The dextran did not migrate significantly in the gel because it lacked any intrinsic charges, thereby representing an upper size limit for an uncharged polysaccharide. In contrast, the supernatant was well resolved in the gel providing further evidence for the presence of significantly charged polysaccharides. The two species with larger size or lower charge-to-size ratio seem to be enriched in the supernatant after filtration, whereas the most abundant species is readily detected in the filtrate. These results are in agreement with the observation that birch #C contains two ice nucleators (see Fig. 1 in main article) and that the ice-binding molecules are much more abundant than the ice-nucleating molecules (see discussion of Fig. 6c in main article).

## S2. Supplementary Methods

**S2.1. Sample preparation for studying the effects of borate addition.** Ice affinity-purified birch pollen washing water samples of birch pollen batch #C (IAP2) were used for the ice nucleation DSC experiments and ice shaping experiments. $Na_2B_4O_7 \cdot 10H_2O$ was added until borate saturation was reached. Diluted samples of birch pollen washing water were used in both cases to allow for complete complexation of saccharide hydroxyl groups by borate due to its moderate solubility in water ($Na_2B_4O_7 \cdot 10H_2O$ solubility at 25 °C: ~60 g $kg^{-1}$ water).[25] These solutions were then stirred overnight at room temperature to ensure a complete reaction.

**S2.2. Sample preparation for studying the effects of pH.** The same ice affinity-purified birch sample was used as that for the experiments with borate, see previous section. For studying the effect of pH on ice nucleation 100 µL of aqueous 0.1 M HCl was added to 1 mL a diluted birch molecule stock solution to obtain a pH value of 2. In order to avoid dilution effects when comparing to ice nucleation in the original stock solution, 100 µL of pure water was added to another 1 mL of stock solution. Moreover, we also performed a DSC experiment with a re-neutralized sample at pH8, which was obtained by adding 100 µL of 0.1 M NaOH to the acidified pH2 sample mentioned above. The effect of pH on ice shaping activity was investigated by adding 10 µL of aqueous 1 M HCl solution to 100 µL of a highly concentrated birch sample to obtain a final pH value of 1.

**S2.3. Fluorophore-Assisted Carbohydrate Electrophoresis.** For the fluorophore-assisted carbohydrate electrophoresis (FACE), the original standard procedure of Goubet et al. was modified.[21] 20 µL of a 0.1 M solution of 8-aminonaphthalene-1,3,6-trisulfonate (ANTS) in acetic acid/water (3:17, v:v) and 20 µL of a 0.5 M $NaCNBH_3$ solution in DMSO were added to 1-2 mg of the freeze-dried oligosaccharides. The reagents were mixed, centrifuged and then incubated at 37 °C for 18 h. Afterwards, the solution was freeze-dried. Finally, 100 µL of a 6 M solution of urea in water was added to the derivatised sugars.

For the gel electrophoresis, a VWR Midi PAGE system with 10 x 10 cm plates and a 1 mm spacer was used. The acrylamide gel (20% w:v) was prepared by adding 4 mL of an acrylamide/N,N'-methylenebisacrylamide solution (30 wt% / 0.8 wt%; Roth) to 1975 µL of 0.1 M Tris-borate buffer, pH 8.2, 5 µL of tetramethylenediamine (TEMED, Sigma) and 20 µL of ammoniumperoxodisulfate (AMPS, 10 wt% in water). After polymerization for 1 h, a stacking gel of 1 cm was cast using 1066 µL of the acrylamide/N,N'-methylenebisacrylamide solution, 2900 µL of buffer, 6 µL of TEMED and 30 µL of

AMPS solutions and polymerized for another hour. 20 µL of the derivatised sugars and 20 µL of a 0.5 µM solution of a maltodextrin ladder labelled with 9-aminopyrene-1,3,6-trisulfonic acid (Glyko GKSP-503, Prozyme) were loaded onto the gel and electrophoresed at 200 V for 20 min and at 500 V for 25 min.

The derivatised sugars were detected after separation using a FUJIFILM Luminescent Image Analyzer LAS-3000 with an excitation wavelength of 460 nm. Here, we studied a once ice-affinity purified (IAP1) sample of birch pollen batch #C, more precisely the stock solution, the 100 kDa supernatant without any washing steps, and the corresponding filtrate. As a reference, an equivalently pre-treated 150 kDa dextran (Fluka Analytical) was studied.

**S2.4. $CaCl_2$ gelation experiments.** To investigate the gel formation ability of the birch pollen polysaccharides 50 µL of a 2 M calcium chloride solution were added to 150 µL of a twice ice affinity-purified and up-concentrated birch pollen sample (IAP2 #C). For comparison, 150 µL of a 0.01 wt% sodium alginate solution was prepared in an analogous manner.

## Supplementary References


1. Budke, C. & Koop, T. BINARY: an optical freezing array for assessing temperature and time dependence of heterogeneous ice nucleation. *Atmos. Meas. Tech.* **8,** 689–703 (2015).

2. Augustin, S. *et al.* Immersion freezing of birch pollen washing water. *Atmos. Chem. Phys.* **13,** 10989–11003 (2013).

3. O'Sullivan, D. *et al.* The relevance of nanoscale biological fragments for ice nucleation in clouds. *Sci. Rep.* **5,** 8082 (2015).

4. Zobrist, B., Marcolli, C., Peter, T. & Koop, T. Heterogeneous ice nucleation in aqueous solutions: the role of water activity. *J. Phys. Chem. A* **112,** 3965–75 (2008).

5. Knopf, D. A. & Forrester, S. M. Freezing of water and aqueous NaCl droplets coated by organic monolayers as a function of surfactant properties and water activity. *J. Phys. Chem. A* **115,** 5579–91 (2011).

6. Knopf, D. A., Alpert, P. A., Wang, B. & Aller, J. Y. Stimulation of ice nucleation by marine diatoms. *Nat. Geosci.* **4,** 88–90 (2010).

7. Koop, T. & Zobrist, B. Parameterizations for ice nucleation in biological and atmospheric systems. *Phys. Chem. Chem. Phys.* **11,** 10839–50 (2009).

8. Knopf, D. A. & Alpert, P. A. A water activity based model of heterogeneous ice nucleation kinetics for freezing of water and aqueous solution droplets. *Faraday Discuss.* **165,** 513–534 (2013).

9. Koop, T., Kapilashrami, A., Molina, L. T. & Molina, M. J. Phase transitions of sea-salt/water mixtures at low temperatures: Implications for ozone chemistry in the polar marine boundary layer. *J. Geophys. Res.* **105,** 26393 (2000).

10. Koop, T., Luo, B., Tsias, A. & Peter, T. Water activity as the determinant for homogeneous ice nucleation in aqueous solutions. *Nature* **406,** 611–4 (2000).

11. Koop, T. Homogeneous Ice Nucleation in Water and Aqueous Solutions. *Zeitschrift für Phys. Chemie* **218,** 1231–1258 (2004).

12. Zobrist, B., Marcolli, C., Pedernera, D. A. & Koop, T. Do atmospheric aerosols form glasses? *Atmos. Chem. Phys.* **8,** 5221–5244 (2008).

13. Clegg, S. L., Brimblecombe, P. & Wexler, A. S. Thermodynamic Model of the System H+ − NH4+ − SO42- − NO3- − H2O at Tropospheric Temperatures. *J. Phys. Chem. A* **102,** 2137–2154 (1998).

14. Friese, E. & Ebel, A. Temperature Dependent Thermodynamic Model of the System H+−NH4+−Na+−SO42−−NO3−−Cl−−H2O. *J. Phys. Chem. A* **114,** 11595–11631 (2010).

15. Carslaw, K. S., Clegg, S. L. & Brimblecombe, P. A Thermodynamic Model of the System HCl-HNO3-H2SO4-H2O, Including Solubilities of HBr, from <200 to 328 K. *J. Phys. Chem.* **99,** 11557–11574 (1995).

16. Isbell, H. S., Brewster, J. F., Holt, N. B. & Frush, H. L. Behavior of certain sugars and sugar alcohols in the presence of tetraborates - correlation of optical rotation and compound formation. *J. Res. Natl. Bur. Stand. (1934).* **40,** 129 (1948).

17. Neven, L. G., Duman, J. G., Low, M. G., Sehl, L. C. & Castellino, F. J. Purification and characterization of an insect hemolymph lipoprotein ice nucleator: evidence for the importance of phosphatidylinositol and apolipoprotein in the ice nucleator activity. *J. Comp. Physiol. B* **159,** 71–82 (1989).

18. Pummer, B. G., Bauer, H., Bernardi, J., Bleicher, S. & Grothe, H. Suspendable macromolecules are responsible for ice nucleation activity of birch and conifer pollen. *Atmos. Chem. Phys.* **12,** 2541–2550 (2012).

19. Pummer, B. G. *et al.* Ice nucleation by water-soluble macromolecules. *Atmos. Chem. Phys.* **15,** 4077–4091 (2015).

20. Goubet, F., Morriswood, B. & Dupree, P. Analysis of methylated and unmethylated polygalacturonic acid structure by polysaccharide analysis using carbohydrate gel electrophoresis. *Anal. Biochem.* **321,** 174–182 (2003).

21. Goubet, F., Dupree, P. & Salomon Johansen, K. in *The Plant Cell Wall: Methods in Molecular Biology* (ed. Popper, Z. A.) **715,** 81–92 (Springer, 2011).

22. Kosik, O., Bromley, J. R., Busse-Wicher, M., Zhang, Z. & Dupree, P. in *Methods in Enzymology: Cellulases* (ed. Gilbert, H. J.) **510,** 51–67 (Elsevier, 2012).

23. Jackson, P. The use of polyacrylamide-gel electrophoresis for the high-resolution separation of reducing saccharides labelled with the fluorophore 8-aminonaphthalene-1,3,6-trisulphonic acid. Detection of picomolar quantities by an imaging system based on a cooled cha. *Biochem. J.* **270,** 705–713 (1990).

24. Tawada, A. *et al.* Large-scale preparation, purification, and characterization of hyaluronan oligosaccharides from 4-mers to 52-mers. *Glycobiology* **12,** 421–426 (2002).

25. Lide, D. R. *CRC Handbook of Chemistry and Physics*. (CRC Press, 2005).

26. Antson, A. A. *et al.* Understanding the mechanism of ice binding by type III antifreeze proteins. *J. Mol. Biol.* **305,** 875–89 (2001).

**Supplementary Figure S1. Cumulative number of ice nucleators per birch pollen grain, $n_n(T)$, versus temperature.** $n_n(T)$ was obtained from BINARY experiments (see methods) with suspensions and washing water of two different batches of birch pollen: batch #A and batch #B show, respectively, two and one heterogeneous ice nucleation signals in DSC experiments (see Fig. 1 of the main article). Data of $n_n(T)$ from suspension (green points) and washing water (blue points) of batch #A agree well with each other, and so do those of washing water of batch #B (red points). At low temperatures (below about –27 °C) all data curves approach a value of $n_n(T) \approx 10^4$, indicating that each pollen grain releases about $10^4$ ice-nucleating molecules, in agreement with previous measurements.[2,3]

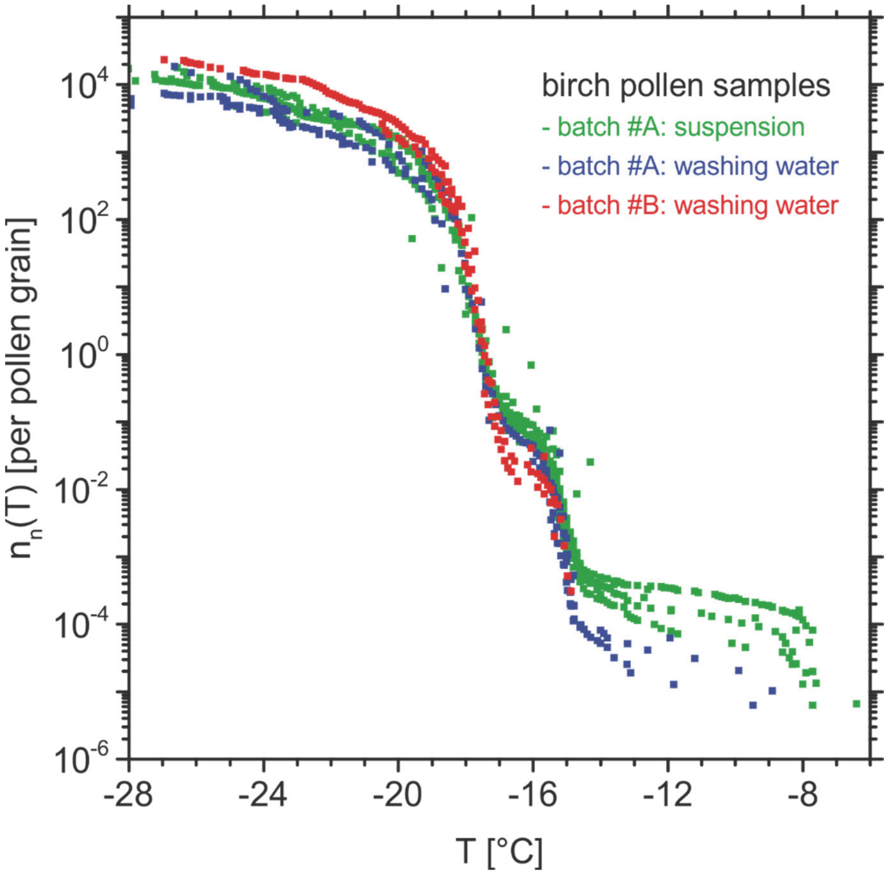

**Supplementary Figure S2. Homogeneous and heterogeneous ice nucleation as well as ice melting temperatures for various emulsified aqueous solutions.** (**a**) DSC thermograms of emulsified samples of pure water (black) and of birch pollen INM in water (red) and in aqueous NaCl solutions of different concentration (green, blue, and orange). (**b**) Data of ice melting temperatures ($T_m$, black), homogeneous ice nucleation temperatures ($T_{hom}$, blue) and two heterogeneous ice nucleation temperatures ($T_{het1}$ and $T_{het2}$, red and green) obtained in DSC experiments are shown as a function of solute mass fraction for NaCl (diamonds), glucose (circles) and NaCl/glucose mixtures (squares). These solutes were mixed with washing water from birch pollen containing ice-nucleating molecules. (**c**) The same data as in (**b**) but as a function of total solute molality. (**d**) The $T_m$, $T_{hom}$, $T_{het1}$, and $T_{het2}$ data from (**a**) and (**b**) plotted as a function of the solution water activity.

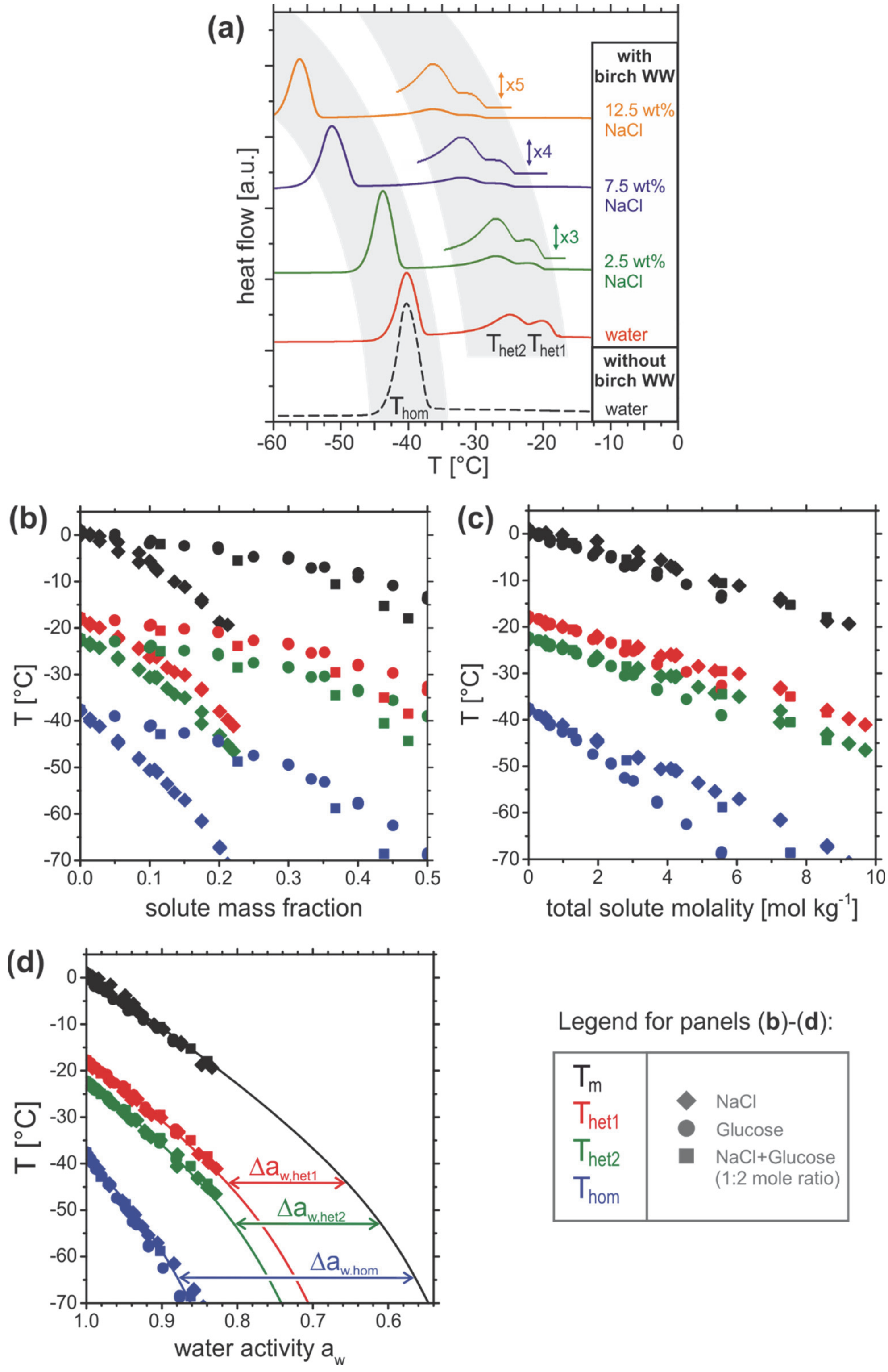

**Supplementary Figure S3. Gelation experiments.** An ice affinity-purified sample of birch pollen #C (IAP2) is a clear solution (**a**), but after addition of aqueous calcium chloride small floccules form (**b**), due to gel formation. For comparison, an alginate solution (0.01 wt%) also forms floccules after addition of aqueous calcium chloride (**c**).

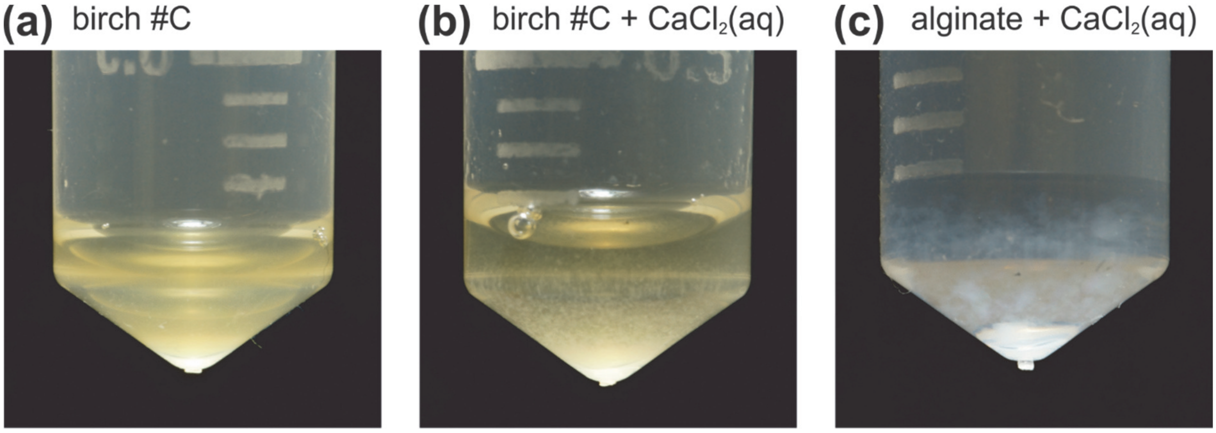

**Supplementary Figure S4. Centrifugal filtration of ice-affinity purified sample of birch pollen batch #B (IAP2) through a 100 kDa cut-off filter.** (**a**) DSC thermograms show the loss of INA in the filtrate sample (bottom panel), while the supernatant (middle panel) shows the same ice nucleation activity as the stock solution (top panel). (**b**) Ice shaping experiments show contrary behaviour. (**c**) FTIR spectra of the corresponding dried residues (spectra are identical to those in Fig. 6c of the main article).

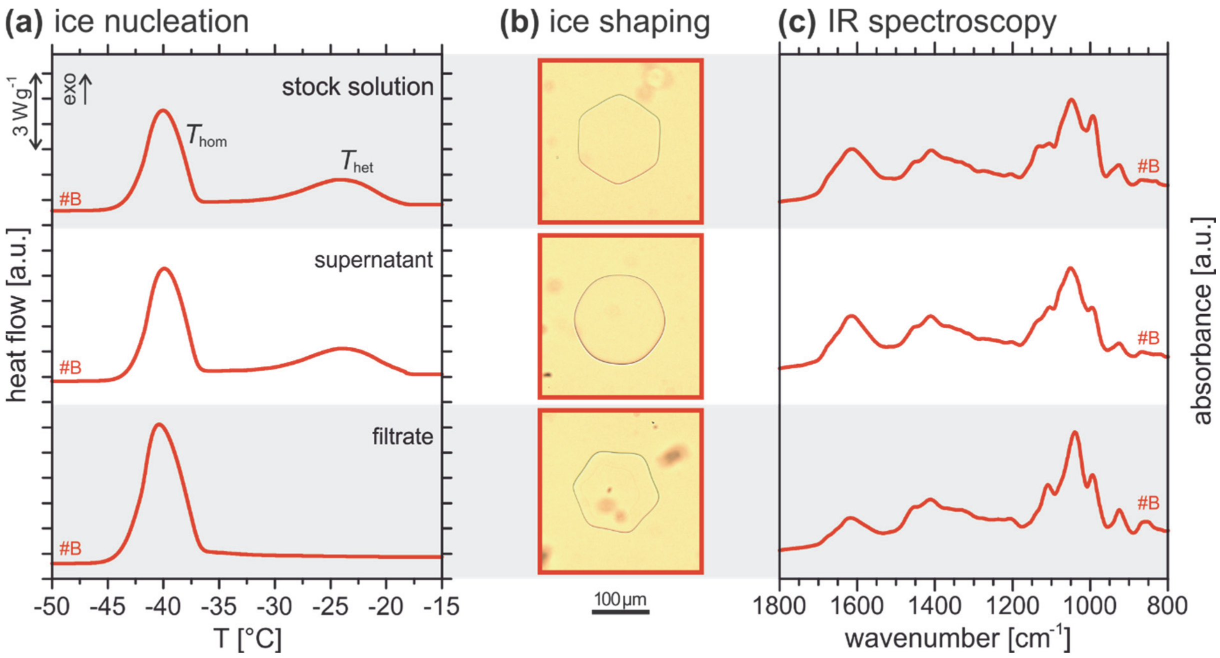

**Supplementary Figure S5. Results of Fluorophore-Assisted Carbohydrate Electrophoresis of birch pollen batch #C washing water before and after ice affinity purification and filtration. (a)** The gel electrophoresis reveals one abundant and at least two minor species of defined size and charge-to-size ratio (indicated by arrows). In the flow-through at the bottom, the unreacted, triply charged fluorophore is observed. The standard is composed of a series of originally uncharged oligomeric maltodextrins. **(b)** A 150 kDa dextran does not penetrate into the gel because of a lack of charges. In contrast, the supernatant of the filtration with a 100 kDa cut-off is well resolved implying the presence of significantly charged polysaccharides. The abundant component also dominates the filtrate.

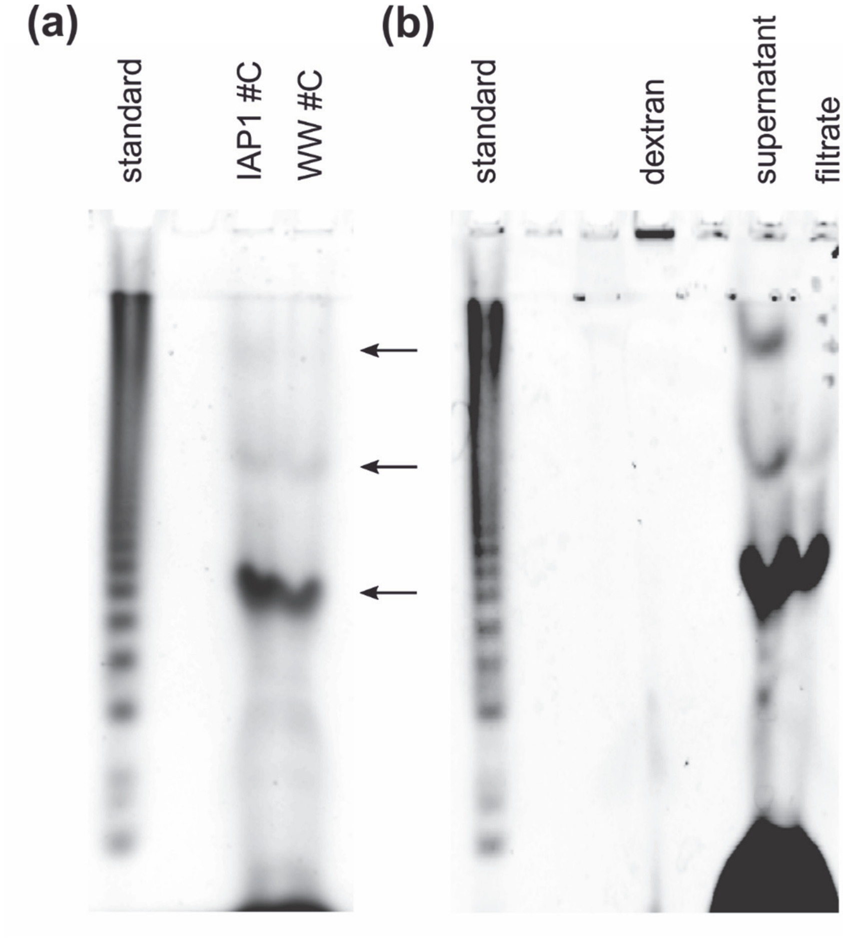

**Supplementary Figure S6. Effect of borate addition and of pH on ice nucleation and ice shaping of twice ice-affinity purified birch pollen sample #C (IAP2).** (**a**) Addition of borate (green line) removes ice-nucleation activity of diluted original birch #C (purple line). (**b**) Minimal ice-shaping activity of a moderately concentrated original #C sample (purple) disappears after addition of borate (green). (**c**) The onset of heterogeneous ice nucleation ($T_{het}$) in diluted original birch #C sample (brown line) is not affected by addition of dilute HCl to yield pH2, but the overall peak size is reduced (red line). Re-neutralization with dilute NaOH to pH8 maintains the ice onset temperature, but does not recover overall peak size (blue line); for details see text in Supplementary Note. (**d**) A 'flower-like' shaping of ice crystals in a highly concentrated original #C sample is not affected by addition of HCl to yield pH1.

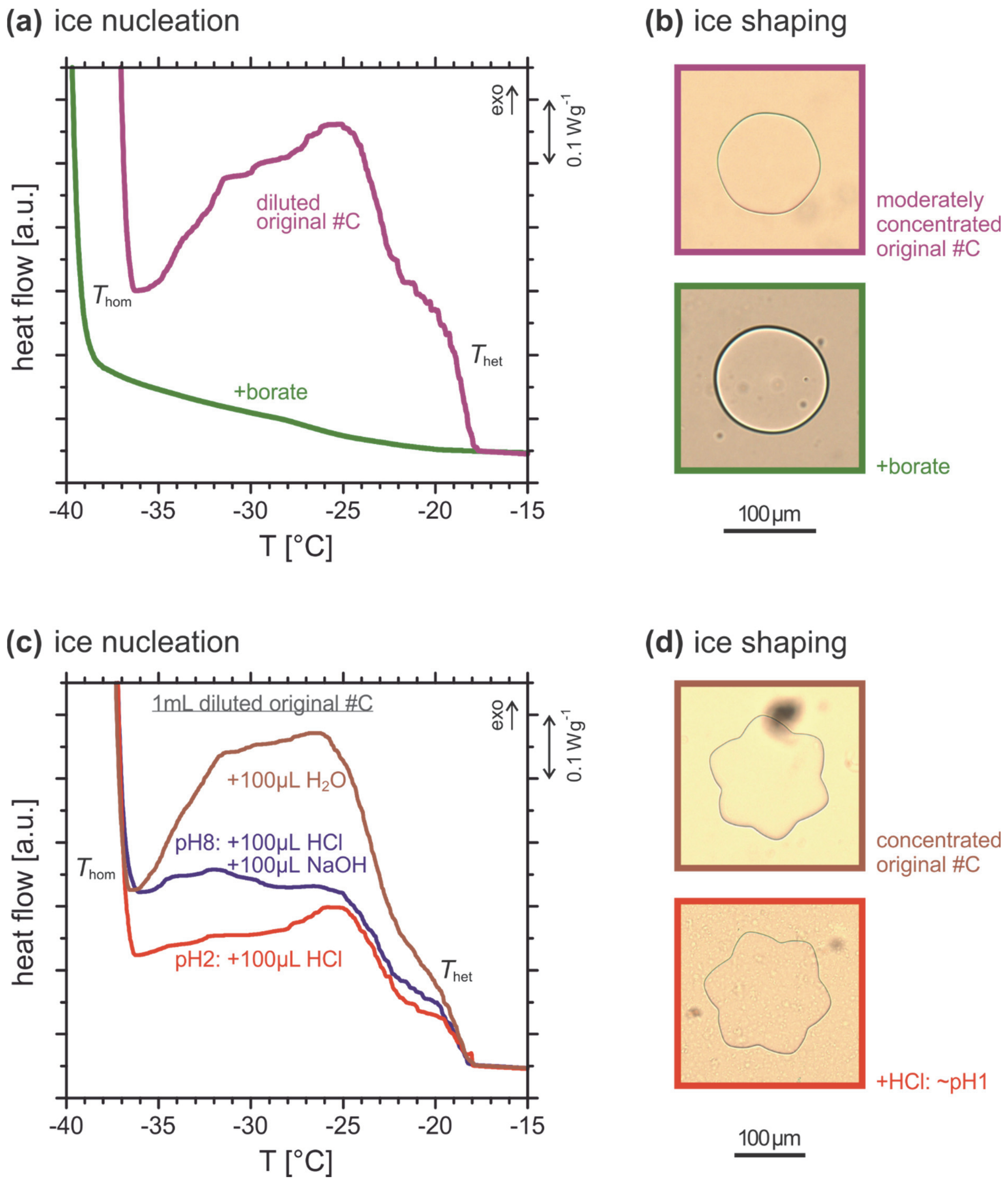

**Supplementary Video S1. Shaping of an ice crystal by birch pollen polysaccharides.** Time-lapse video of a single ice crystal growing in an aqueous sucrose solution containing ice-binding birch pollen polysaccharides purified by two cycles of ice affinity purification. See sequence of still images in Fig. 3c of the main article for comparison. Supplementary Video S1 accompanies the online version of the manuscript and is available for download in the corresponding section 'Supplementary Information'.

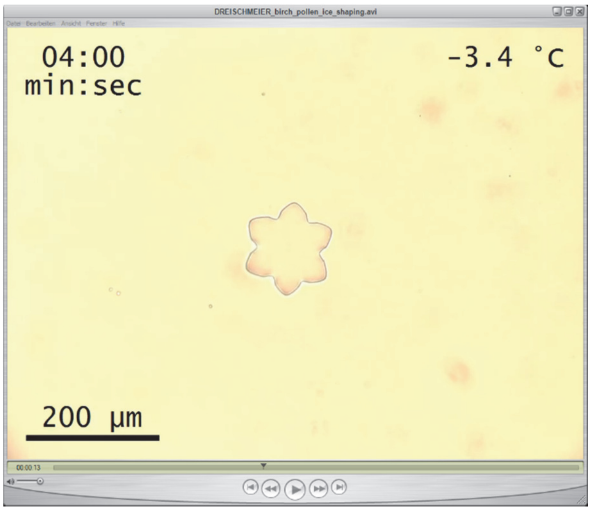

**Supplementary Table S1. Chemical structure of reference compounds used in FTIR experiments.** The colour coding agrees with that of the spectra shown in Fig. 5b of the main article.

| Name | CAS-Number | Chemical Formula and Structure |
|---|---|---|
| **Hyaluronate Sodium Salt** | 9067-32-7 | $(C_{14}H_{20}NO_{11})_n$ 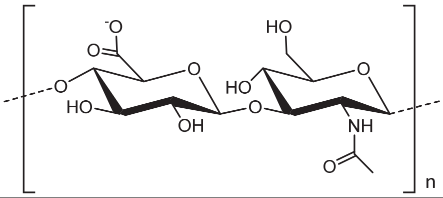 <br> Acidified: hyaluronic acid $(C_{14}H_{21}NO_{11})_n$ 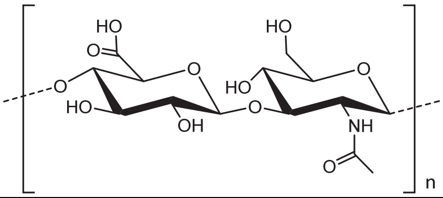 |
| **Cellulose** | 9004-34-6 | $(C_{12}H_{20}O_{10})_n$ 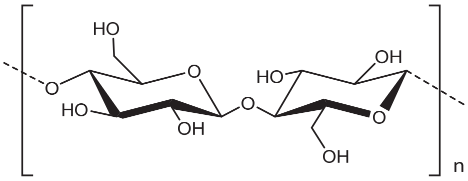 |
| **Dextran** | 9004-54-0 | $(C_6H_{10}O_5)_m$ (randomly branched polymer) 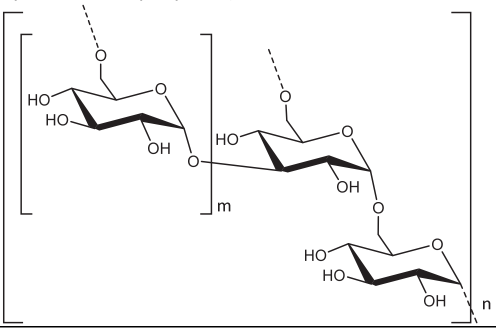 |
| **AFP III** | PDB-ID: 1hg7 | Protein (for amino acid sequence, see Antson et al.[26]) 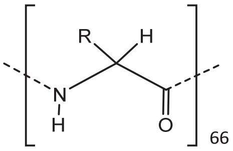 |
| **Alginate Sodium Salt** | 9005-38-3 | $(C_6H_7O_6)_m(C_6H_7O_6)_n$ 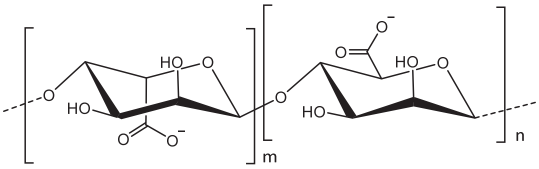 |
| **Heparin Sodium Salt** | 9041-08-1 | Sulphated glycosaminoglycan with repeating unit D-glucosamine and D-glucuronic acid or L-iduronic acid; many hydroxyl and amino groups are sulphated 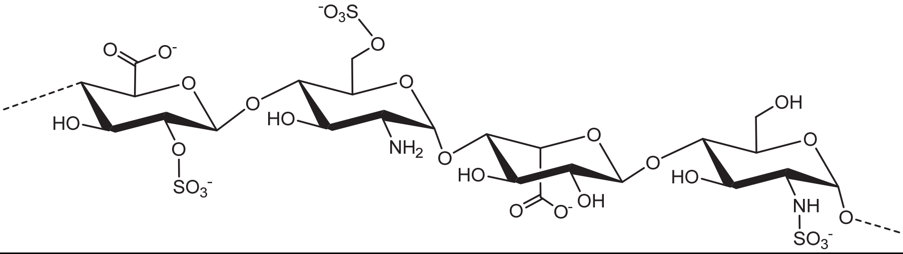 |

**Supplementary Table S2. Coefficients for the parameterization of the water activity of aqueous solutions of glucose and NaCl according to Supplementary Equation (S1).** The parameterization for NaCl is applicable only to NaCl mass fractions of *w* = 0.0-0.23 and temperatures of *T* = 263.15-293.15 K, but it was extrapolated to lower temperatures in the current work.

| solute | a | b | c | d | e | f | g |
|---|---|---|---|---|---|---|---|
| glucose | −1 | −0.91879 | 0.082547 | $4.3113 \cdot 10^{-4}$ | $-5.7258 \cdot 10^{-5}$ | $-3.7372 \cdot 10^{-4}$ | 0 |
| NaCl | −2.14909 | −1.60295 | −0.01239 | $9.01426 \cdot 10^{-4}$ | −0.0373 | 0.21138 | −0.37088 |
